# Non-Visual Photopigments Effects of Constant Light-Emitting Diode Light Exposure on the Inner Retina of Wistar Rats

**DOI:** 10.3389/fneur.2017.00417

**Published:** 2017-08-21

**Authors:** María M. Benedetto, Mario E. Guido, María A. Contin

**Affiliations:** ^1^Facultad de Ciencias Químicas, Departamento de Química Biológica “Dr. Ranwel Caputto”, Universidad Nacional de Córdoba, Córdoba, Argentina; ^2^Centro de Investigaciones en Química Biológica de Córdoba (CIQUIBIC), CONICET, Universidad Nacional de Córdoba, Córdoba, Argentina

**Keywords:** retinal degeneration models, changes in retinal structure, non-visual opsin localization, light-emitting diode light, retinal light damage

## Abstract

The retina is part of the central nervous system specially adapted to capture light photons and transmit this information to the brain through photosensitive retinal cells involved in visual and non-visual activities. However, excessive light exposure may accelerate genetic retinal diseases or induce photoreceptor cell (PRC) death, finally leading to retinal degeneration (RD). Light pollution (LP) caused by the characteristic use of artificial light in modern day life may accelerate degenerative diseases or promote RD and circadian desynchrony. We have developed a working model to study RD mechanisms in a low light environment using light-emitting diode (LED) sources, at constant or long exposure times under LP conditions. The mechanism of PRC death is still not fully understood. Our main goal is to study the biochemical mechanisms of RD. We have previously demonstrated that constant light (LL) exposure to white LED produces a significant reduction in the outer nuclear layer (ONL) by classical PRC death after 7 days of LL exposure. The PRCs showed TUNEL-positive labeling and a caspase-3-independent mechanism of cell death. Here, we investigate whether constant LED exposure affects the inner-retinal organization and structure, cell survival and the expression of photopigments; in particular we look into whether constant LED exposure causes the death of retinal ganglion cells (RGCs), of intrinsically photosensitive RGCs (ipRGCs), or of other inner-retinal cells. *Wistar* rats exposed to 200 lx of LED for 2 to 8 days (LL 2 and LL 8) were processed for histological and protein. The results show no differences in the number of nucleus or TUNEL positive RGCs nor inner structural damage in any of LL groups studied, indicating that LL exposure affects ONL but does not produce RGC death. However, the photopigments melanopsin (OPN4) and neuropsin (OPN5) expressed in the inner retina were seen to modify their localization and expression during LL exposure. Our findings suggest that constant light during several days produces retinal remodeling and ONL cell death as well as significant changes in opsin expression in the inner nuclear layer.

## Introduction

The retina is the neural portion of the eye adapted to capturing light photons and transmitting this information to other structures in the brain. In mammals, light acts directly in the retina to fulfill two important roles: the visual function through rods and cones photoreceptor cells (PRCs) and the so-called “non-image forming tasks” such as the photoentrainment of circadian rhythms, pupilary light response, melatonin secretion, sleep regulation, and light-dependent relaxation of the retinal vasculature. These tasks are carried out by intrinsically photosensitive RGCs (ipRGCs), a subset of RGC that project to the suprachiasmatic nucleus in the hypothalamus and other non-visual areas (1–3). These cells, identified for the first time in the dermal melanophores of *Xenopus laevis* (4), express the photopigment melanopsin (OPN4) and thus respond directly to light. In the ipRGCs of mammalian and non-mammalian vertebrates that express OPN4, light causes cell depolarization, triggering trains of action potentials during the stimuli (1). These cells exhibit a phototransduction cascade similar to that observed in invertebrate photoreceptors with calcium mobilization, activation of phospholipase C and the phosphoinositide cycle, as well as of the transient receptor potential channels (5–10). It is known that OPN4-expressing ipRGCs are dysfunctional in various retinal and optic nerve diseases (11) and that they may be more resistant to injury than the general RGC population (12).

In addition to OPN4, the inner retina may express other photopigments and photoisomerases such as encephalopsin/panopsin (OPN3), neuropsin (OPN5), peropsin, retinal G protein coupled receptor (RGR), vertebrate ancient opsin, and cone opsins (13–18).

OPN5 protein has been identified in the ganglion cell layer (GCL) of *Wistar* rat retinas (19), indicating that it may be a novel class of PRC. It is a UV-sensitive (λ max 380 nm) Gi-coupled non-visual opsin, and is reported to drive entrainment of retinal circadian oscillators to light dark cycles independently of rods, cones and OPN4 (20). Furthermore, OPN5 mediates extraretinal photoreception in the cornea and perhaps other tissues as well (21).

Light exposure is a well characterized path toward retinal degeneration (RD) (22–28). Excessive exposure to light as a consequence of light pollution (LP) caused by environmental artificial lighting may accelerate degenerative diseases or promote RD and circadian desynchrony in healthy patients. The effects of artificial lighting and its strong impact on environmental degradation (29–31) is underestimated in today’s world, as are the potential consequences for human health, through circadian alterations and retinal diseases (32). Furthermore, new devices containing light emitting diodes (LEDs) have a high component of blue light (wavelengths of 460–500 nm), known to play a major role in RD (33–38) and possibly to activate the non-visual system, including ipRGCs.

Excessive exposure to light is a rapidly growing problem in current society and retinal diseases will become a major problem in decades to come. Studies on long-term exposure to low levels of blue light are required in order to determine potential effects on the eye. To this end, we investigated the molecular mechanisms underlying RD and the putative role of artificial LED sources in these mechanisms. We consider that a better understanding of the molecular mechanisms triggered by low light intensity exposure leading to RD could provide valuable insights into the progression of clinical disorders related to phototransduction defects caused by mutations in related proteins, macular degeneration or the LP effect. Previously, in our laboratory, we evaluated the time course and molecular mechanisms of RD in *Wistar* rats during continuous low-intensity LED light exposure. We found that contant light, but not cyclic exposure to 200 lx for 8 days, produces cell death in PRC rods but does not alter rhodopsin expression before cell death. However, the photopigment from LL animals was found to be more phosphorylated in ser^334^ than in controls, supporting the notion that changes in the regulation of the phototransduction cascade occur during RD (28). Our goal in the present work is to determine whether the inner retina is affected by LED sources. To this end, we examine light effects on the inner retina, specifically in the RGC (ipRGCs) and inner nuclear layer (INL) cells, as well as on the expression and localization of OPN4 and OPN5. No visible death in RGC or INL cells was recorded at any time studied in Wistar rats stimulated by constant exposure to 200 lx of diffuse LED light for several days; however, at the level of opsin expression in the inner retina, we found significant changes in the localization and protein expression for both opsins after LL exposure. Our findings indicate that constant light exposure produces retinal remodeling with cell death in the outer nuclear layer (ONL) and a relocation of photopigments responsible for sensing light in the inner retina.

## Materials and Methods

### Animals

All animal procedures were performed in accordance with the ARVO statement for the use of animals in ophthalmic and vision research, which was approved by the local animal committee (School of Chemistry, UNC, Exp. 2013–291). Male adult *Wistar* rats, inbred in our laboratory for 40 years, aged 12–15 weeks were exposed to a 12:12 h light dark cycle (LD); with the light on (less than 50 lx of fluorescent lamp) from zeitgeber time (ZT) 0 to 12 for all animals from the time they were born up to the experiment. Food and water were available *ad libitum*.

### Retinal Light Damage

**Figure 1:**
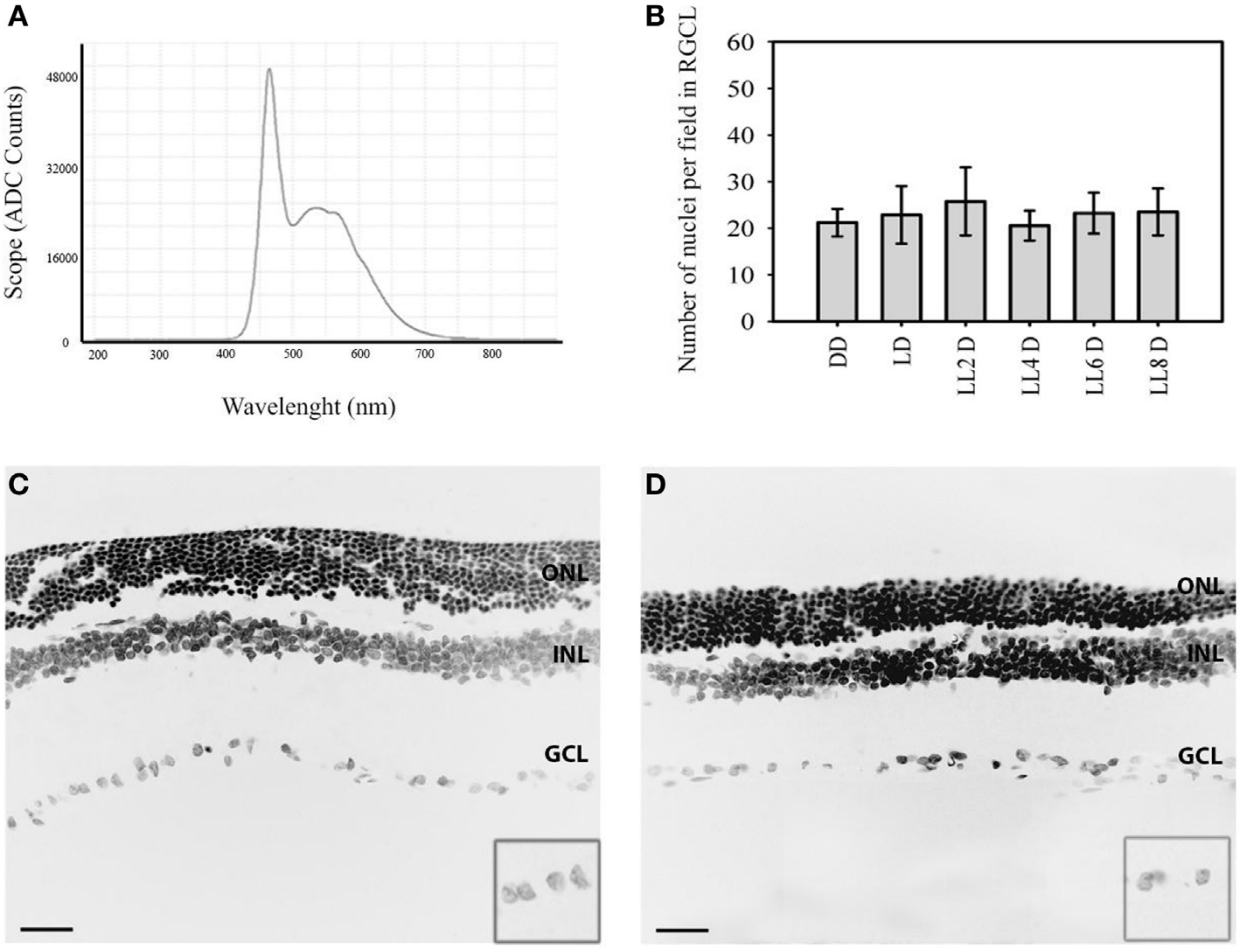
Representative nuclear staining in retinal images in responses to LL treatment: **(A)** spectral distribution of white light-emitting diode light source. **(B)** Quantification of retinal ganglion cell nuclei at DD (constant dark), LD 8, LL 2, LL 4, LL 6, LL 8 days. **(C,D)** Representatives retinas showing DAPI staining in LD8 control, Wistar rats maintained in LD cycle for 8 days **(C)** and LL8-treated Wistar rats maintained in LL during 8 days **(D)**. Data are mean ± SD (*n* = 1 animal/group) from five independent experiments, *p* > 0.05 by a non-parametric Kruskal–Wallis ANOVA. Insert, magnification of GCL. Barr 10 µm. ONL, outer nuclear layer; INL, inner nuclear layer; GCL, ganglion cell layer.

Retinal degeneration was induced in a temperature-controlled light stress box equipped with 200 lx of diffuse, cool white LED light (EVERLIGHT Electronic Co., Ltd., T-13/4 3294-15/T2C9-1HMB) fixed on the inner upper surface, which illuminated the interior white walls. The spectrum distribution of LED light source was tested using a spectrometer (AvaSpec-HS1024x58/122TEC- Avantes BV, The Netherlands, Europe) and was expressed as scope vs. wavelength (nanometers) (Figure 1A). The illumination at the level of the rats’ eyes was measured as 200 lx with a light meter (model 401036; Extech Instruments Corp., Waltham, MA, USA). At midday (ZT 6), all rats were killed in a CO_2_ chamber after 2, 4, 6, and 8 days after constant light stimulation (LL); light dark cycle (0 lx: 200 lx, LD) or constant dark (DD) according to the experimental design (*n* = 4 at each point). Control experiments were performed by exposing all animals to 200 lx under a 12:12 h LD, with light on at ZT 0 and off at ZT 12.

### Histological and Quantitative RGC Analysis

The methods employed for fixation, embedment, sectioning, and histological analysis of eyes were as previously described (32). Briefly, rat eyes at different days after light or control treatment were fixed in 4% (W/V) paraformaldehyde in 100 mM [phosphate buffer saline (PBS), pH 7.3] overnight at 4°C, before being cryoprotected in sucrose and mounted in an optimal counting temperature compound (OCT; Tissue-Tek^®^ Sakura). Retinal sections 10-µm thick were then cut along the horizontal meridian (nasal-temporal) using a cryostat (Cryostat HM525 NX-Thermo Scientific). The sections were fixed for 30 min in 4% paraformaldehyde in PBS and cover slips were washed in PBS, treated with blocking buffer (PBS supplemented with 0.1% BSA, 0.1% Tween 20, and 0.1% NaNO3), and incubated two overnight at 4°C with Opn4 antibody (ab 19306 Abcam dilution: 1/500 v/v) or Opn5 (NOVUS biologicals, Littleton, CO, USA, Cat. NB110-74726) antibody in the incubation buffer as described (9, 39, 40). They were then rinsed in PBS and incubated with goat anti-rabbit IgG Alexa Fluor 546 (monoclonal antibody) or monoclonal mouse anti-rabbit IgG antibody (1:1,000) (Jackson immunoresearch) for 1 h at room temperature (RT). Samples were subsequently incubated with DAPI (2.18 mM). Cover slips were finally washed thoroughly and visualized by confocal microscopy (FV1000; Olympus, Tokyo, Japan).

To quantify RGC the sections were lightly stained with DAPI (final concentration: 3 µM) for 10 min and photographed using a confocal microscope (Olympus FV1200, Japan) at 40× or 60× magnification. The number of ganglion cell nuclei was counted on the photograph of each of the four designated retinal areas—left, middle left, middle right, and right (for 10 different animals per treatment). Quantitative ganglion cells analysis was performed as (28) to quantify cell survival (width and length of the micrograph), using the software ImageJ (v. 1.45), and the plugin “Automatic Nuclei Counter.”

### Retinal Whole Mount

Retinal whole mount immunohistochemist was performed in a similar manner, except that isolated retinas instead of whole eyecups were processed for staining, and the retinas were incubated in primary antibodies for 48 h.

### TUNEL Staining

The terminal deoxynucleotidyl transferase dUTP nick end labeling (TUNEL) assay was performed following the procedures provided with the kit from Roche Diagnostics Corp. (Indianapolis). Briefly, frozen sections of rat retinas were cut on a cryostat before being postfixed with 4% paraformaldehyde and permeabilized in PBS, 0.1% (W/V) sodium citrate, 0.1% (V/V) Triton X-100. The reaction mixture (50 µl TUNEL) was added to each sample and the slides were incubated in a humidified atmosphere for 60 min at 37°C in the dark. For negative controls, the TdT enzyme was replaced by label solution, and the samples from three different animals per treatment were analyzed by a confocal microscope (Olympus FV300, Tokyo, Japan).

### Western Blot

Homogenates of whole rat retina resuspended in 200 µl PBS buffer containing the protease inhibitor cocktail were lysed by repeated cycles of ultra-sonication, and the total protein content was determined by the Bradford method (41). As an OPN5 positive control, homogenates of mouse ear were processed as in rat (42). The homogenates of whole rat retina were then resuspended in sample buffer [62.5 mM Tris–HCl pH 6.8; 2% (W/V) SDS; 10% (V/V) glycerol; 50 mM DTT; 0.1% (W/V) bromophenol blue] and heated at 90°C for 5 min. The proteins (20 µg) and molecular weight marker [5 µl ECL Rainbow Marker-Full range (12,000–225,000 Da) from Amersham Code RPN7800E] were separated by SDS-gel electrophoresis on 12% polyacrylamide gels, transferred onto nitrocellulose membranes, blocked for 1 h at RT with 5% (W/V) skim milk in PBS, and then incubated overnight at 4°C with OPN4 antibody (ab 19306 Abcam dilution: 1/500 v/v) or OPN5 antibody [NOVUS biologicals; Littleton, CO, 80160, USA; (Cat. number: NB110- 74726)] in the incubation buffer. The membranes were subsequently washed, incubated with the secondary antibody goat anti-rabbit IDye 680, and scanned in the Odyssey IR Imager. Six retinas from three independent experiments were analyzed for each time point. Densitometry of western blots was performed using the ImageJ software and the ratio from the quantitative analysis of three oligomer band optical densities in LL relative to LD was expressed as relative expression.

### Statistical Analysis

Data are expressed as mean ± SD. Statistical comparisons were made using one-way ANOVA; *p*-value < 0.05 was considered statistically significant. To prove the normality and homogeneity of the variance assumption, Shapiro–Wilks and Levene tests were used. In all cases with significance, a Dunkan or Tukey *post hoc* test with a *p* value < 0.05 was considered statistically significant. In cases of non-parametric analysis, the Kruskal–Wallis test was conducted.

## Results

### Quantitative RGC Analysis

In order to determine the light stimuli effects on the inner retina, *Wistar* rats were exposed to 200 lx of white LED light without pupil dilatation for the indicated times; the spectrum distribution of LED light source shows the higher component of blue light (480 nm) compared with the other wavelengths of visible light (Figure 1A). The analysis of retinal sections for the overall thinning in LD controls (Figure 1C) or light-treated retina (Figure 1D) shows a notable reduction in the ONL nucleus after 8 days of light stimuli; however, the number of RGCs does not change along the days of stimuli. The quantitative analysis of the number of RGC nuclei does not reveal significant differences between different light exposure groups (LL2-8) with respect to controls kept in DD or LD (Figure 1B). This result indicates that LL exposure at 200 lx with white LED does not produce RGC death.

### TUNEL Assay and Structural Analysis

**Figure 2:**
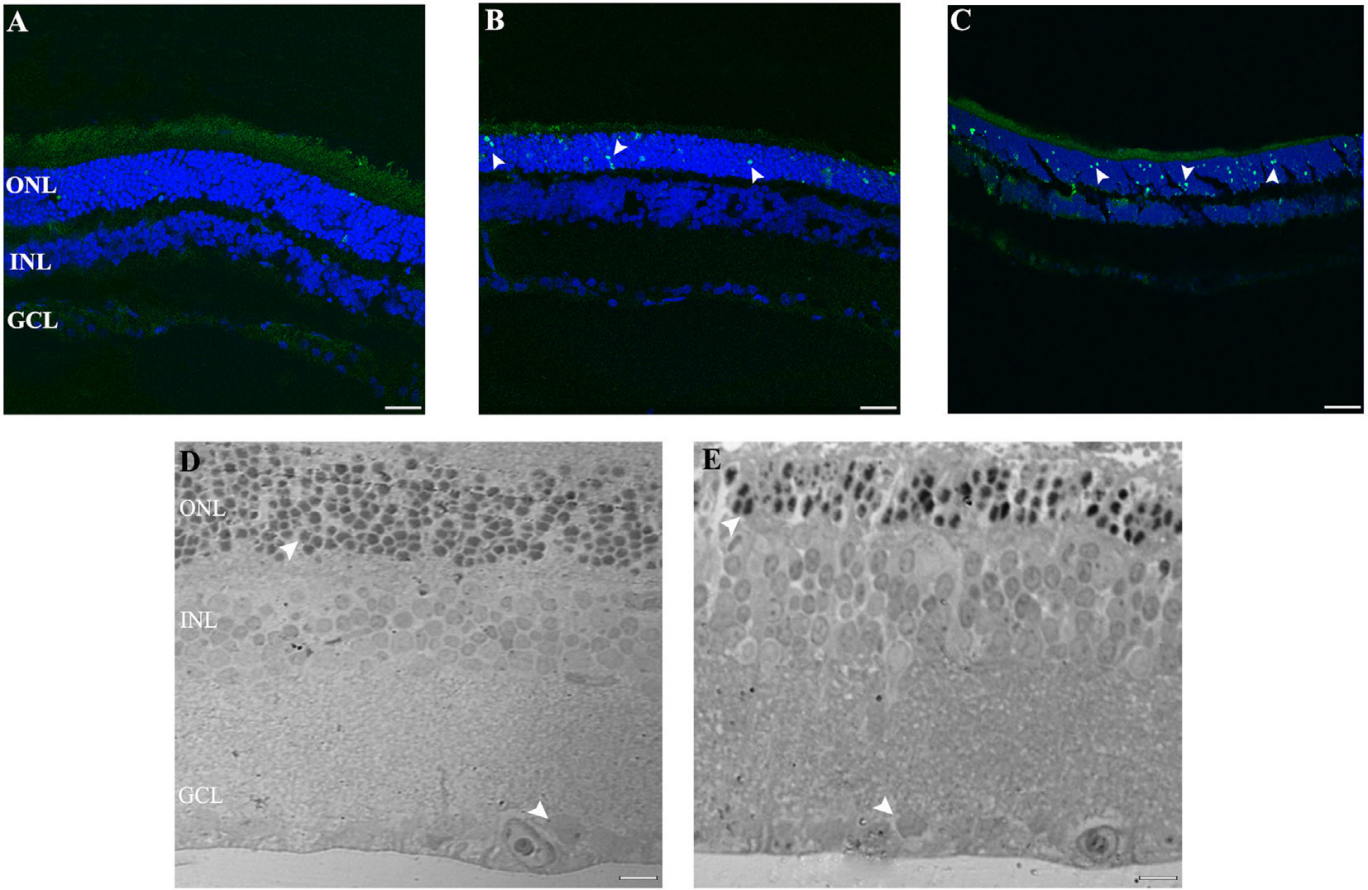
Cell death study in retinal light damage: **(A–C)** TUNEL staining at 2, 4, and 8 days of LL treatment, arrowheads show the TUNEL positive in the ONL. **(D,E)** Transmittance images of entire width of retina maintained in LD **(D)** and LL8 treated **(E)**, showing a clear reduction in ONL after 8 days of LL stimuli; normal **(D)** and pyknotic nuclei **(E)** in the ONL and normal nuclei in GCL **(D,E)**. Scale bar indicates 30 µm. ONL, outer nuclear layer; INL, inner nuclear layer; GCL, ganglion cell layer.

To further assess whether RGCs are subject to the process of cell death at any time of light exposure tested, we analyzed DNA fragmentation using the TUNEL assay. Previously, we showed positive TUNEL ONL staining in the retinas of rats exposed to 4 days of LL (28), whereas no staining was detected after 4 days in retinas in the LD conditions, indicating that the thinning of the retina induced by light exposure was caused by PRC death. We then analyzed the effects of LL stimuli on RGC survival. Figure 2 shows a representative experiment of a TUNEL assay performed to assess cell death in the entire retina, at different times of light exposure. As can be observed, there are no TUNEL-positive cells labeled in the GCL and INL at any time of light exposure studied, clearly indicating no cell death in these regions (Figures 2A–C, 2, 4, and 8 of LL, respectively). However, in ONL including classical photoreceptor nuclei, a few TUNEL-positive cells were observed in LL 2, with a significant increase in numbers along the days of light exposure. These findings indicate that despite producing classical PRC death, low exposure to white LED light does not produce the death of INL cells and RGC.

The ultrastructural analysis of transmittance confocal microscopy shows a largely intact morphology after LL 8 in the inner retina (INL and GCL) (Figures 2D,E, filled rows). However, unlike in the LD controls, many pyknotic nuclei could be seen after LL 8 in ONL; the nuclei appeared misaligned and the outer segment structure was disrupted, indicating that ONL is the only nuclear layer affected by light treatment.

### OPN4 Analysis

**Figure 3:**
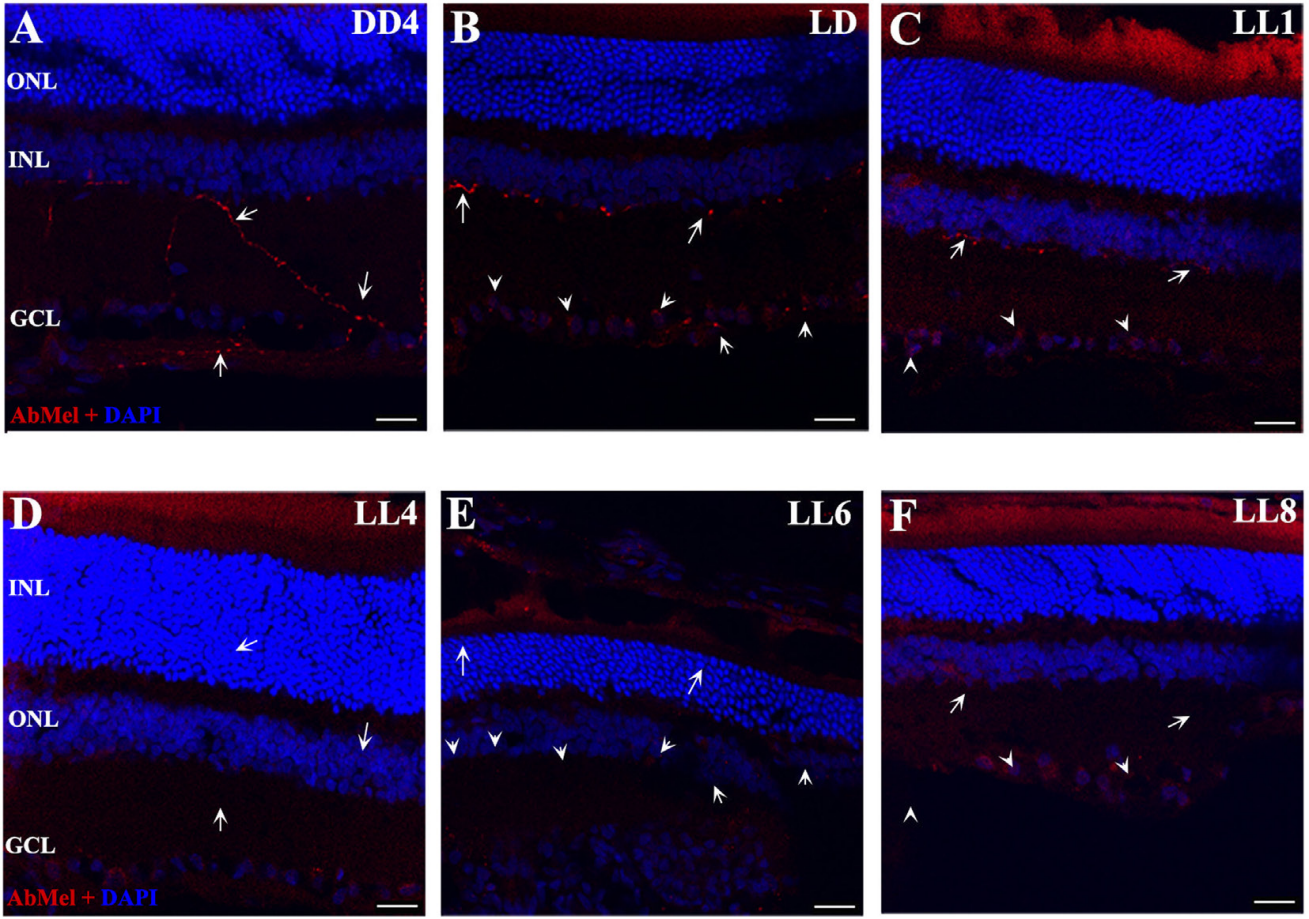
OPN4 expression in retinal cuts of animals exposed to light: **(A)** retinas maintained in dark during 4 days showing positive labeling along the dendrites which extend into INL. **(B)** Retinas maintained in LD 8 showing a pattern similar to that in DD. **(C–F)** Light-treated animals during 1, 4, 6, and 8 days in LL showing increasing label restricted to cell soma around nuclear area. Arrow shows the positive labeling of OPN4 in soma, axons, and dendrites. The images are representative of three different experiments per treatment. Red: OPN4 antibody staining; blue: nuclear DAPI staining. Scale bar indicates 30 µm.

**Figure 4:**
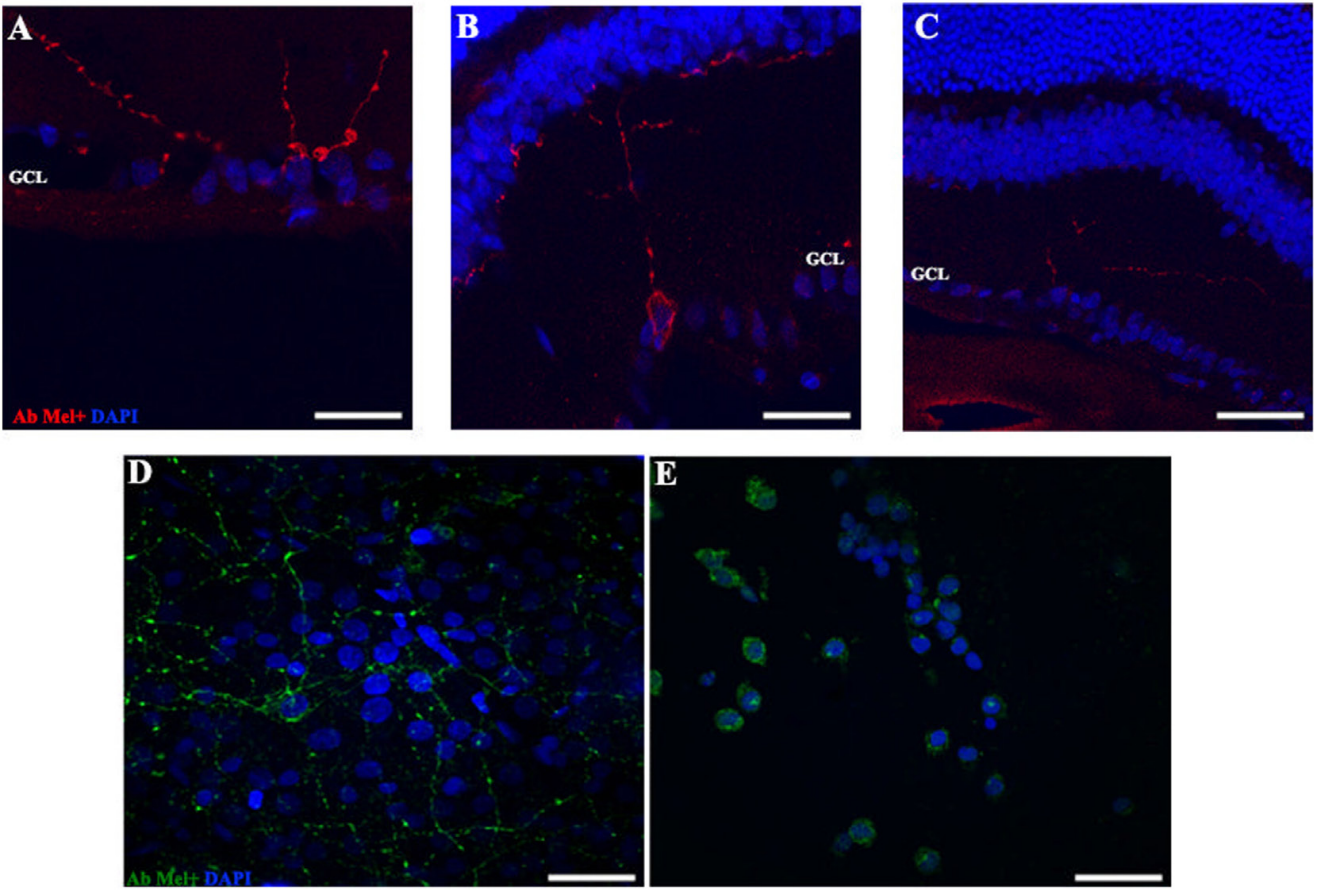
OPN4 expression in retinal cuts and whole mount in animals exposed to light: details of retinas maintained in dark during 4 days **(A)**; LD 8 days **(B)** or 8 days at LL **(C)** at higher magnification than in Figure 3. Red: OPN4 antibody staining; blue: nuclear DAPI staining. Scale bar indicates 10 µm. **(D,E)** stained retinal whole mount with retinal ganglion cell side up of retina from animals maintained in LD **(D)** and LL 8 days **(E)** showing a differential distribution of OPN4 labeling. Blue: DAPI, green: OPN4 antibody. The images are representative of three different experiments per treatment. Scale bar indicates 30 µm.

In order to determine whether the population of ipRGCs is affected by LL stimuli, retinas from animals subject to different numbers of days of LL exposure were processed to assess OPN4 expression, as indicated in the Section “Materials and Methods.” Figure 3 shows that OPN4 immunostaining changes with time of light exposure. On LL 1, OPN4 appears similar to the control (Figures 3A–C), along the dendrites which extend into INL; however, after LL 6 and LL 8, OPN4 staining was only visible in the somas of RGCs, showing a visible increase in immunostaining (Figures 3D–F) that clearly indicates a possible internalization or higher expression in cell somas. Detailed analysis of the images taken from light-exposed or control retinas clearly shows differential distribution between controls in LD and LL 8 exposed animals (Figures 4A–C). The analysis of the entire retina by retinal whole mount analysis shows a clear distribution in neuronal cells with little staining in the soma zone in the area corresponding to RGCs during the first days; however, after 8 days of light exposure (LL 8) the OPN4 label is restricted to cell soma around the nuclear area (Figures 4D,E).

**Figure 5:**
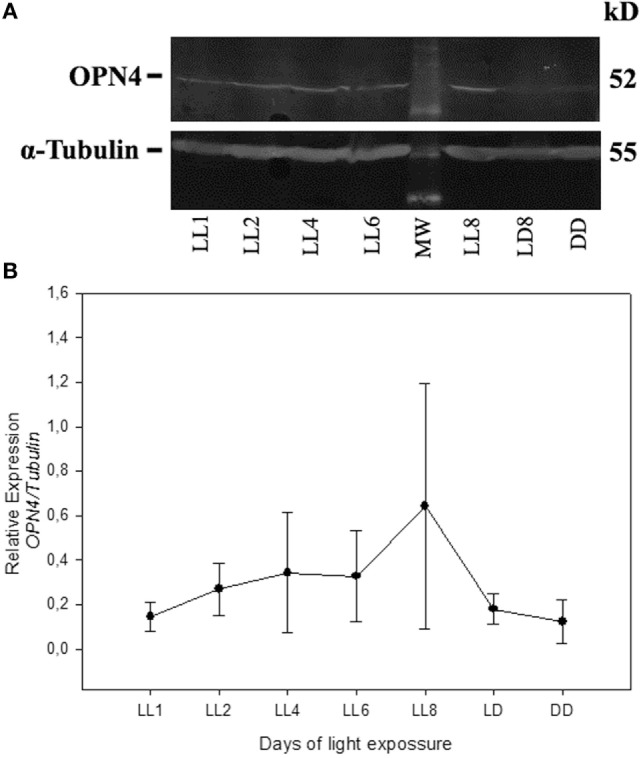
Analysis of Western blot of OPN4 protein expression in retina of animals exposed to light: **(A)** western blot immunolabeled OPN4 with a specific antibody showing the predicted isoform bands at different times of light treatment (LL, 1, 2, 4, 6, and 8 days) and controls in LD and DD during 8 days. The margin shows the molecular mass (right). **(B)** Relative expression of OPN4 normalized to α-Tubulin. Data are mean ± SD from three independent experiments; *p < 0.05 vs. DD4 by a non-parametric Kruskal–Wallis ANOVA.

In order to detect any possible changes in the expression of this photopigment, in addition to the redistribution study, we quantified the OPN4 protein expression by western blot. As shown in Figure 5, the protein analysis indicates a slight increase in the level of expression during LL exposure, with higher levels at LL 8. The quantitative analysis of densitometry immunostaining shows only statistical significance (*p* < 0.05) between DD and LL 8 (Figure 5B). These results indicate that although the RGCs do not exhibit significant levels of cell death, the photopigment OPN4 in the ipRGC subpopulation presents different localization within the cell body and processes with a slight increase in protein expression.

### OPN5 Analysis

**Figure 6:**
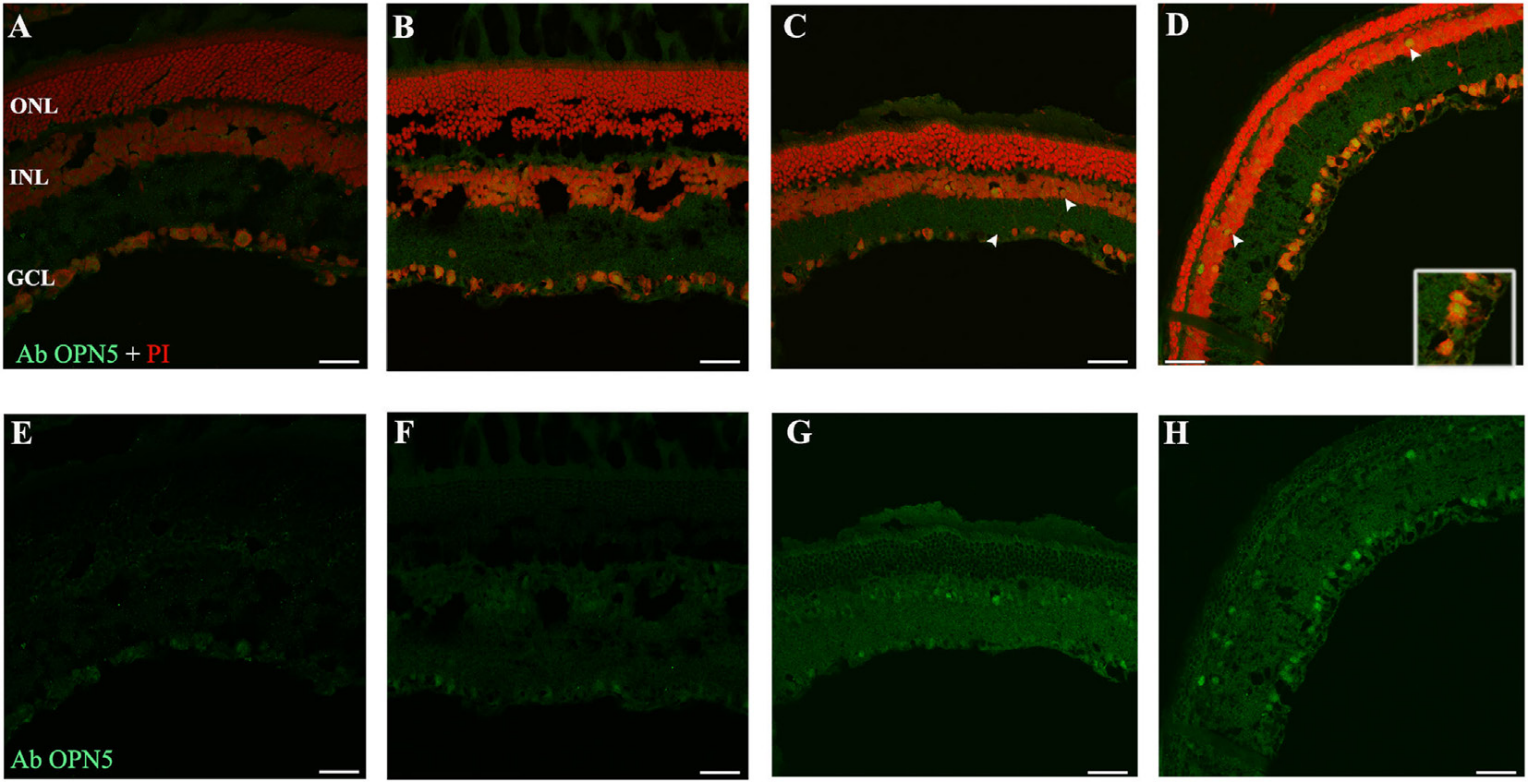
Analysis of OPN5 protein expression in retina of animals exposed to light: **(A,E)** retinas maintained in dark during 4 days showing low positive staining in retinal ganglion cell (RGC). **(B,F)** Retinas maintained in LD 8 days showing a similar pattern as in DD. **(C–H)** Light-treated animals during 2 and 8 days in LL showing labeling in RGC and INL. The images are representative of three different experiments per treatment. Green: OPN4 antibody staining; blue: nuclear PI staining. Scale bar indicates 30 µm.

As reported previously, OPN5 was observed in the INL of *Wistar* rat retina (40), suggesting that the presence of OPN5 in the GCL of rat retina may indicate the existence of another kind of PRC in the inner retina. Here, we study the light exposure effects on the localization and expression of this protein. As shown in Figure 6, low levels of OPN5 immunostaining were seen in some GCL cells in DD and LD controls (Figures 6A,B, respectively); however, the levels of opsin expression increased in animals exposed to a higher number of days of LL, reaching the highest levels of expression at LL8 (Figures 6C,D, LL4 and LL 8, respectively). Furthermore, OPN5 is also observed in other cells corresponding to the INL area from LL4 and LL 8 (see arrows).

**Figure 7:**
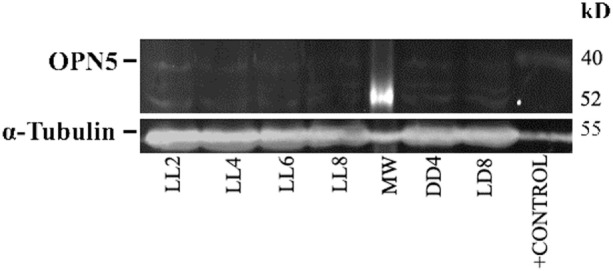
Analysis of western blot of OPN5 protein expression in retina of animals exposed to light: western blot immunolabeled OPN5 with a specific antibody showing the predicted isoform bands at different times of light treatment (LL, 2, 4, 6, and 8 days) and controls in light dark and DD during 8 days. The margin shows the band corresponding to OPN5 and β-tubulin (left) and the molecular mass markers (right). Positive control: rat ear homogenate. The images are representative of three different experiments per treatment.

In order to detect any possible changes in the expression of this photopigment, we quantified the OPN5 protein expression by western blot. As shown in Figure 7, the protein analysis indicates a slight increase in the level of expression during LL exposure, with higher levels at LL 8. The quantitative analysis of protein levels did not reveal any statistical differences between LL treatment with respect to controls, suggesting that LL stimuli produce a slight increase in OPN5 protein in the inner retina, likely in GCL and some INL cells.

## Discussion

Light pollution constitutes a growing problem worldwide. The consequences of excessive artificial illumination in modern day life may not only accelerate degenerative diseases or promote RD and circadian desynchrony in healthy patients but will also have a strong impact on the vision of future generations. The working model presented in this paper provides a useful tool to study specific events associated with RD under conditions of low light with LED sources, at constant or enduring periods of exposure (LP context). Previously, we demonstrated that PRCs died along the days of continuous light stimuli with a significant reduction in ONL at LL7 (28). Furthermore, in electroretinographical studies, we found that both in temporal and frequency analyses, the alterations caused by the light stimuli become noticeable after 5 days of LL; LL4 could represent a point of inflexion, where the retinal cells, mainly photoreceptors, show a statistically significant dysfunction in both the time domain and frequency (43), indicating that the process of RD occurs along the first 7 days of LL. In this work, we studied constant light effects in the inner retina with a view to elucidating overall retinal processes and to determine whether inner-retinal cells can be photoresistent. We did not find a significant reduction in the number of RGC nuclei neither by TUNEL staining at the different times of exposure studied (Figures 1 and 2A–C), strongly suggesting that LL exposure does not affect cell survival at this layer. The structural analysis by transmittance microscopy in retinas from animals exposed to LL for 8 days reveals a clear reduction in the width of the entire retina and a lower number of nuclei in ONL, many with a pyknotic appearance, than in LD treated animals (Figures 2D,E). However, the area in the IPL and INL appears to be normal, without pyknotic nuclei and with blood vessels of normal appearance (see rows). We speculate that overall events promoted by light effects may induce deep retinal remodeling. Longer LL exposure times likely cause some inner-retinal damage as a secondary effect, providing the opportunity to study the mechanisms of inner processes during the time lapse described here. Different RD models of prolonged light exposure cause rod and cone death in albino rodents, which clearly may involve multiple components including rhodopsin bleaching, RPE cell death, abnormal retinoid processing and oxidative reactive species (44) and as a secondary effect, inner-retinal damage. We are interested in studying the biochemical events taking place in these processes during the progress of RD. It is important to note that this is the first paper to report that constant light exposure does not produce RGC death. It is known that cell death and retinal remodeling involving RGC occurs in inherited retinal disorders (45–47). In albino rats has been demonstrated that constant exposure to high intensity light metabolically stresses RGCs, triggering their death (44, 48–50). Sang et al. (48) described a retinal light damage model with RGC dying at high levels of light stimuli (light wavelength ranges from 400 to 760 nm, 8,000 lx for 3 h) in pupil dilated, dark-adapted male Sprague–Dawley rats (48). In addition, the upregulation of a Pyruvate kinase isozyme type M2, a key glycolytic enzyme involved in multiple cellular processes including translocation to the nucleus, thus inducing cell apoptosis, indicates an association with the mechanisms of RGC apoptosis (51). The upregulation of Fra-1 protein was also shown to be associated with RGC apoptosis (52), indicating the occurrence of RGC death in the RD process. These findings suggest that the light condition used may produce different mechanisms of RD and specific effects in different retinal cell populations. Despite the apparent visual normality of the internal retina at LL 1–8, changes occur in the expression of OPN4 and OPN5 and they relocalize. For OPN4, LL shows decreased levels of immunoreactivity in distal neurite processes, but slight increases in cell soma, suggesting that light exposure produces a reduction in OPN4 levels in distal regions and an increase by *de novo* synthesis in cell soma, or by transport from the distal region to cell soma, or both (Figures 3 and 4). This increase is further supported by western blot analysis (Figure 5). These observations cannot be attributed to a higher concentration of ganglion cell proteins as a result of a significant reduction in protein classic photoreceptors (rods and cones), since Thy-1 expression did not change at any time studied (result none shown). We may infer that this pigment relocalization could fulfill the lower requirements of sensitivity in the presence of LL compared with the requirements of retinas exposed to LD or DD; or that due to the fact that the signal input from the outer retina is atrophied in light-treated animals, the retina requires a remodeling process with the relocalization of OPN4. Although many studies in recent years have demonstrated that the expression of OPN4 is regulated by the effects of light, the functional implications of this are not yet clear. Wong et al. (53) provided evidence that ipRGCs undergo light and dark adaptation independently of the lighting conditions. It is known that OPN4 expression is regulated by the circadian clock (54–58); however, prolonged periods of LL exposure have a suppressing effect on OPN4 levels (55, 56, 59). In *Wistar* rats exposed to >300 lx of white light, OPN4 expression is upregulated during prolonged darkness and sluggishly downregulated in prolonged light (protein expression), showing by means of immunohistochemistry an extensive dendrite network (56) which seems to override the clock-controlled regulation of OPN4. Our observations partially differ from the above-mentioned studies in that here we demonstrate decreased levels of immunoreactivity in distal dendrites or axons, but increased levels of protein expression in cell soma. There are at least two possible explanations for these discrepancies: the difference in the results from the two studies may be attributed to differences in the higher intensity of LL exposure used or to the source of illumination used. We stimulate *Wistar* rats with white LED light of 200 lx of intensity which has a higher relative intensity of 480 nm (see Materials and Methods), known to be harmful to classic PRCs (28, 32). OPN4 is sensitive to a range of wavelengths reaching a peak of light absorption at blue-light wavelengths around 488 nm (60). As speculated above, continuous blue-light stimuli by LED may produce OPN4 remodeling changes and have a strong effect on the regulation of circadian rhythms.

In the OPN5 analysis, we demonstrated increasing levels of immunolabeling in some cells of GCL and INL after LL 4 with the highest value observed at LL8. The microscope analysis shows a differential expression during the days of LL exposure with a higher label in perinuclear areas (Figure 6). This indicates that OPN5 is also regulated by the effect of light, showing relocalization and increasing protein expression (Figure 7). Opn5 belongs to an independent group of opsins separate from the other groups in the phylogenetic tree of photopigments and constitutes a functional UV-sensitive Gi-coupled bistable photopigment with maximal efficiency at 420 nm. It is expressed in the deep brain in the hypothalamus of birds (14, 17) and has been immunolocalized to the mammalian inner retina, specifically in GCL cells of the adult retina (40). These signals were not seen when immunostaining was performed using antigen-adsorbed antibodies and the localization of Opn5m in the retina is conserved between mammals and chicken (61). In *Wistar* rats, the expression of mRNA in eye and protein expression specifically within the retina in INL and GCL cells and in IPL processes (19) has been demonstrated. Light-sensing function for mammalian OPN5 is not fully known; Buhr et al. (62) demonstrated that OPN5 is required for the photoentrainment of the local circadian clock of retina which regulates many important functions, such as photoreceptor disk shedding. Changes in the expression and localization of OPN5 by the continuous blue-light stimuli by compensatory effects may affect the regulation of local circadian rhythms in the retina.

## Conclusion

Retinal degeneration involves many complex processes causing tissue dysfunction that can eventually lead to blindness and circadian rhythm desynchrony. Light is an important effector that induces many of these processes either in pathological retinas with some degenerative mutation or in healthy retinas. LP may cause some of all these events. Our results demonstrate that constant light produces specific retinal remodeling within the outer retina accompanied by significant cell death in the ONL as previously reported. By contrast, in the inner retina and cells located at the INL and GCL, continuous light exposure does not appear to severely affect cell survival though a substantial relocation of non-visual photopigments OPN4 and OPN5 -responsible for sensing light in the inner retina- could clearly be observed. This can be understood as a compensatory mechanism to protect against the light damage occurring in the ON.

We speculate that this relocalization of photopigments as part of a compensatory mechanism in the inner retina may produce changes in its general physiology. Variations in the synchronization of circadian rhythms through OPN4 regulation or changes in the inner retina physiology through OPN5, concomitant with the absence of ONL, may produce degeneration of the inner retina as a consequence of longer exposure times to light. Further studies on longer exposure to LL are required in order to test this hypothesis. Overall, the RD model presented here is a useful tool for studying the biochemical and pathological mechanisms taking place under LP conditions in which RGCs and others cells in the inner retina are not subject to immediate death and clearly exhibit survival mechanisms in response to continuous LED light exposure.

## Author Contributions

MB: first author. She did the experiments of the all figures presented here. Contributed with the discussion. MG: he contributed with the redaction and discussion. MC: designed and planned the study and drafted the manuscript, with all authors.

## Conflict of Interest Statement

The authors declare that the research was conducted in the absence of any commercial or financial relationships that could be construed as a potential conflict of interest.
